# Association Between Baseline Echocardiographic Parameters and Acute Coronavirus Disease 2019 Infection in Hospitalized Patients

**DOI:** 10.7759/cureus.55432

**Published:** 2024-03-03

**Authors:** Jin Wang, Dongmei Yang, Cheng Cao

**Affiliations:** 1 Department of Echocardiography, The First Affiliated Hospital of University of Science and Technology of China (USTC), Hefei, CHN; 2 Department of Cardiology, The First Affiliated Hospital of University of Science and Technology of China (USTC), Hefei, CHN

**Keywords:** sars-cov-2 omicron variant, short-term outcomes, left ventricular function, coronavirus disease 2019 (covid-19), doppler echocardiography

## Abstract

Background

The current study aimed to examine the association between baseline clinical and echocardiographic parameters with new-onset coronavirus disease 2019 (COVID-19) infection.

Methodology

We retrospectively enrolled consecutive hospitalized patients from our center during the national outbreak of the COVID-19 pandemic in China. Overall, 100 patients were enrolled, including 38 patients with COVID-19 infection.

Results

Compared with those without infection, patients with COVID-19 infection were more likely male (63.2% vs. 35.5%, p = 0.008), were older (59.08 vs. 52.35 years, p = 0.022), had higher heart failure (31.6% vs. 11.3%, p = 0.018) and hypertension (52.6% vs. 30.6%, p = 0.036) rates, had lower left ventricular ejection fraction (LVEF) (61.16% vs. 65.76%, p = 0.018), had higher A-wave velocity (86.84 vs. 73.63 cm/s, p = 0.003), and had and lower E/A ratio (0.85 vs 1.04, p = 0.015). On univariate and multivariate analysis, baseline echocardiographic parameters (LVEF and A-wave velocity) were independent risk factors for COVID-19 infection. There were no significant changes in echocardiographic parameters during the one-month follow-up period in patients infected and not infected with COVID-19.

Conclusions

In conclusion, baseline echocardiographic parameters were significantly associated with acute COVID-19 infection.

## Introduction

Since December 2019, the coronavirus disease 2019 (COVID-19) pandemic has significantly impacted human life. Detrimental prognosis in COVID-19 involves patient demographics and cardiovascular risk factors and diseases, such as older age, high blood pressure, and heart failure [1-3]. Cardiac injury is common in patients with COVID-19 infection and contributes to poor outcomes [4]. Transthoracic echocardiography is widely used, and early studies showed that clinical and subclinical cardiac impairment analyzed by echocardiography was common in COVID-19 patients [5-10]. These studies were mainly performed among patients who were already infected with or recovered from COVID-19 [8-11]. However, the differences in baseline clinical and echocardiographic parameters between patients infected and not infected with COVID-19 remain unclear, especially for the Omicron variant. After the outbreak of the COVID-19 pandemic, China strongly controlled the disease, with restrictions gradually relaxed in November 2022 [12,13]. Following this, the number of patients infected with COVID-19 increased sharply and contributed to the nationwide outbreak of the COVID-19 pandemic (the Omicron variant wave) in China [12,13]. During this period, people were exposed to COVID-19 at the same time and most patients were infected with COVID-19 for the first time. In this study, we sought to determine the association between baseline clinical and echocardiographic parameters with acute COVID-19 infection among hospitalized patients in our center during this national outbreak.

## Materials and methods

**Figure 1:**
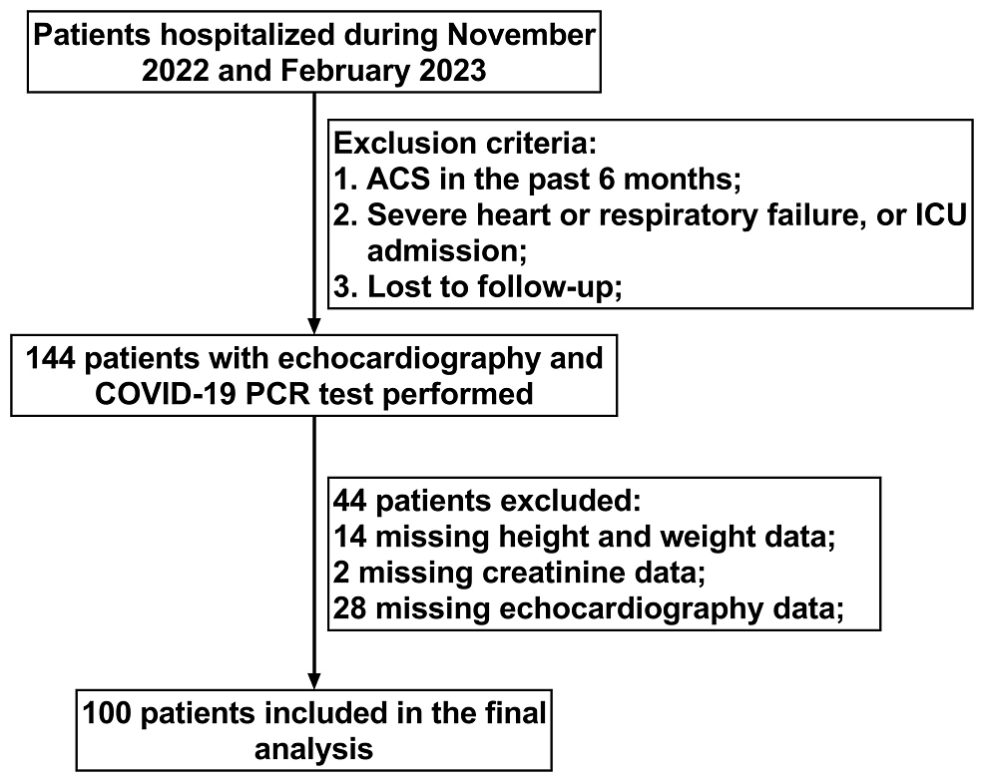
Flowchart of the study. ACS = acute coronary syndrome; ICU = intensive care unit; COVID-19 = coronavirus disease 2019; PCR = polymerase chain reaction

We retrospectively enrolled consecutive patients hospitalized in our center from November 2022 to February 2023. This was the nationwide outbreak period of COVID-19 infection in China, during which most people were infected with COVID-19 for the first time [12,13]. The inclusion criteria were (1) patients aged 18 years and older; (2) patients for whom echocardiography was performed; and (3) patients for whom the COVID-19 test was performed by polymerase chain reaction assay. The exclusion criteria were (1) patients with acute coronary syndrome in the past six months before enrollment; (2) patients with severe heart failure (patients with New York Heart Association grade IV), respiratory failure (patients with respiratory support), or those admitted to the intensive care unit; and (3) patients lost to follow-up. The flow chart of the study is shown in Figure 1. The study was performed in accordance with the Declaration of Helsinki and was approved by the hospital ethics committee (approval number: 2023-RE-085).

Clinical data were obtained from the hospital’s electronic medical system. Body mass index (BMI) was calculated using weight (kg) divided by the square of height (m). The modified Modification of Diet in Renal Disease formula was used to evaluate the estimated glomerular filtration rate [14].

Transthoracic echocardiography was conducted in accordance with the recommendations of previous studies [15]. Images were taken from standardized views and measured by a single experienced sonographer blinded to patients’ clinical data. Relative wall thickness (RWT) and left ventricular mass index (LVMI) were evaluated, as described previously [16]. Stroke volume index and cardiac index were calculated by indexed stroke volume and cardiac output to body surface area, respectively. Body surface area was calculated, as described previously [16]. Echocardiography date from around 1 month after enrollment were collected.

The continuous variables were presented as mean ± standard error (SE) or median value ± interquartile range and were analyzed using the t-test or Mann-Whitney U test, as appropriate. The categorical variables were presented as numbers (%) and were analyzed with the chi-square test. The logistic regression model was applied to explore the associations of baseline clinical and echocardiographic parameters with acute COVID-19 infection. A two-way analysis of variance with the post-hoc test was applied to compare echocardiographic parameters in the enrollment and one-month follow-up period. Statistical analysis was performed using SPSS version 27 (IBM Corp., Armonk, NY, USA) and GraphPad Prism version 9 (GraphPad Software, San Diego, CA, USA). A two-sided p-value <0.05 was considered significant.

## Results

A total of 100 patients were enrolled in the final analysis, including 38 patients with COVID-19 infection (Table 1). Compared with those in the negative group, patients with COVID-19 infection were more likely to be male (63.2% vs. 35.5%, p = 0.008), were older (59.08 vs. 52.35 years, p = 0.022), and had higher heart failure (31.6% vs. 11.3%, p = 0.018) and hypertension (52.6% vs. 30.6%, p = 0.036) rates. Furthermore, patients with COVID-19 infection had lower left ventricular ejection fraction (LVEF) (61.16% vs. 65.76%, p = 0.018), higher A-wave velocity (86.84 vs. 73.63 cm/s, p = 0.003), and lower E/A ratio (0.85 vs 1.04, p = 0.015) compared with patients in the negative group. Moreover, there were no statistically significant differences in lower septal e’-wave velocity (6.77 vs. 7.53 cm/s, p = 0.081) and lower stroke volume index (43.43 vs. 45.78 mL/m^2^, p = 0.087) in patients with COVID-19 infection than those in the negative group. On the other hand, no statistically significant differences in other clinical and echocardiographic parameters were observed between the two groups.

**Table 1:** Baseline clinical and echocardiographic markers between patients infected with COVID-19 and patients in the negative group. COVID-19 = coronavirus disease 2019; GFR = glomerular filtration rate; RWT = relative wall thickness; LVIDd = left ventricular internal diameter in diastole; LVMI = left ventricular mass index; LVEF = left ventricular ejection fraction

	Negative, n = 62	Positive, n = 38	P-value
Clinical characteristics			
Male sex, n (%)	22 (35.5%)	24 (63.2%)	0.008
Age, years	52.35 ± 1.81	59.08 ± 2.22	0.022
Body mass index, kg/m^2^	23.47 (22.62, 24.33)	23.63 (22.02, 25.24)	0.749
Systolic blood pressure, mmHg	122.32 ± 1.86	126.76 ± 2.91	0.181
Diastolic blood pressure, mmHg	79.24 ± 1.39	79.00 ± 1.74	0.914
Heart rate, beat/min	82.18 (79.02, 85.33)	87.29 (82.44, 92.14)	0.108
Estimated GFR, mL/min/1.73 m^2^	113.40 (101.26, 125.54)	97.02 (79.44, 114.59)	0.246
Fasting blood glucose, mg/dl	114.82 (102.77, 126.86)	103.22 (94.79, 111.64)	0.445
Atrial fibrillation, n (%)	4 (6.5%)	3 (7.9%)	1.000
Heart failure, n (%)	7 (11.3%)	12 (31.6%)	0.018
Coronary heart disease, n (%)	12 (19.4%)	8 (21.1%)	1.000
Hypertension, n (%)	19 (30.6%)	20 (52.6%)	0.036
Diabetes, n (%)	9 (14.5%)	10 (26.3%)	0.190
Current smoking, n (%)	11 (17.7%)	5 (13.2%)	0.589
Current alcohol consumption, n (%)	11 (17.7%)	5 (13.2%)	0.589
Echocardiography			
Left atrial diameter, mm	35.92 ± 0.73	37.61 ± 1.15	0.197
RWT, mm	0.39 (0.38, 0.41)	0.40 (0.38, 0.43)	0.288
LVIDd, mm	49.73 (48.53, 50.92)	50.92 (48.25, 53.60)	0.957
LVMI, g/m^2^	106.26 (99.55, 112.97)	112.44 (101.76, 123.13)	0.452
LVEF, %	65.76 (63.38, 68.14)	61.16 (57.01, 65.31)	0.018
E-wave velocity, cm/s	71.23 (66.18, 76.27)	69.68 (62.09, 77.28)	0.469
A-wave velocity, cm/s	73.63 ± 2.43	86.84 ± 3.78	0.003
e’ septal, cm/s	7.53 (6.94, 8.13)	6.77 (6.09, 7.45)	0.081
E/A ratio	1.04 (0.93, 1.14)	0.85 (0.74, 0.96)	0.015
E/e’ ratio	10.26 (9.07, 11.44)	10.99 (9.45, 12.53)	0.350
Stroke volume index, mL/m^2^	45.78 (43.55, 48.00)	43.43 (40.42, 46.43)	0.087
Cardiac index, L/min/m^2^	3.60 (3.42, 3.78)	3.76 (3.42, 4.10)	0.943

Table 2 presents the association between both baseline clinical and echocardiographic characteristics with acute COVID-19 infection. On univariate logistic regression, male sex (odds ratio (OR) = 3.12, 95% confidence interval (CI) = 1.35 to 7.22, p = 0.008), age (OR = 1.04, 95% CI = 1.00 to 1.07, p = 0.026), heart failure (OR = 3.63, 95% CI = 1.28 to 10.28, p = 0.015), hypertension (OR = 2.52, 95% CI = 1.09 to 5.80, p = 0.030), LVEF (OR = 0.96, 95% CI = 0.93 to 1.00, p = 0.049), A-wave velocity (OR = 1.03, 95% CI = 1.01 to 1.05, p = 0.004), and E/A ratio (OR = 0.26, 95% CI = 0.08 to 0.86, p = 0.028) were associated with acute COVID-19 infection. On multivariate logistic regression, only LVEF (OR = 0.96, 95% CI = 0.91 to 1.00, p = 0.041) and A-wave velocity (OR = 1.04, 95% CI = 1.01 to 1.06, p = 0.003) remained significantly associated with acute COVID-19 infection, while male sex (OR = 2.42, 95% CI = 0.98 to 5.98, p = 0.057) was not statistically significant associate with acute COVID-19 infection.

**Table 2:** Univariate and multivariate analysis of baseline clinical and echocardiographic parameters for COVID-19 infection. COVID-19 = coronavirus disease 2019; CI = confidence interval; RWT = relative wall thickness; LVIDd = left ventricular internal diameter in diastole; LVMI = left ventricular mass index; LVEF = left ventricular ejection fraction

	Univariate analysis		Multivariate analysis	
Variable	Odds ratio (95% CI)	P-value	Odds ratio (95% CI)	P-value
Male sex	3.12 (1.35-7.22)	0.008	2.42 (0.98-5.98)	0.057
Age, years	1.04 (1.00-1.07)	0.026	-	
Atrial fibrillation	1.24 (0.26-5.88)	0.784	Not selected	
Heart failure	3.63 (1.28-10.28)	0.015	-	
Coronary heart disease	1.11 (0.41-3.03)	0.837	Not selected	
Hypertension	2.52 (1.09-5.80)	0.030	-	
Diabetes	2.10 (0.77-5.78)	0.149	Not selected	
Left atrial diameter, mm	1.04 (0.98-1.11)	0.198	Not selected	
RWT, mm	19.39 (0.03-14589.26)	0.380	Not selected	
LVIDd, mm	1.03 (0.97-1.10)	0.356	Not selected	
LVMI, g/m^2^	1.01 (0.99-1.02)	0.301	Not selected	
LVEF, %	0.96 (0.93-1.00)	0.049	0.96 (0.91-1.00)	0.041
E wave velocity, cm/s	1.00 (0.98-1.02)	0.721	Not selected	
A wave velocity, cm/s	1.03 (1.01-1.05)	0.004	1.04 (1.01-1.06)	0.003
e’ septal, cm/s	0.85 (0.71-1.03)	0.105	Not selected	
E/A ratio	0.26 (0.08-0.86)	0.028	-	
E/e’ ratio	1.03 (0.95-1.13)	0.453	Not selected	
Stroke volume index, ml/m^2^	0.97 (0.93-1.02)	0.203	Not selected	
Cardiac index, L/min/m^2^	1.25 (0.78-2.03)	0.358	Not selected	

**Figure 2:**
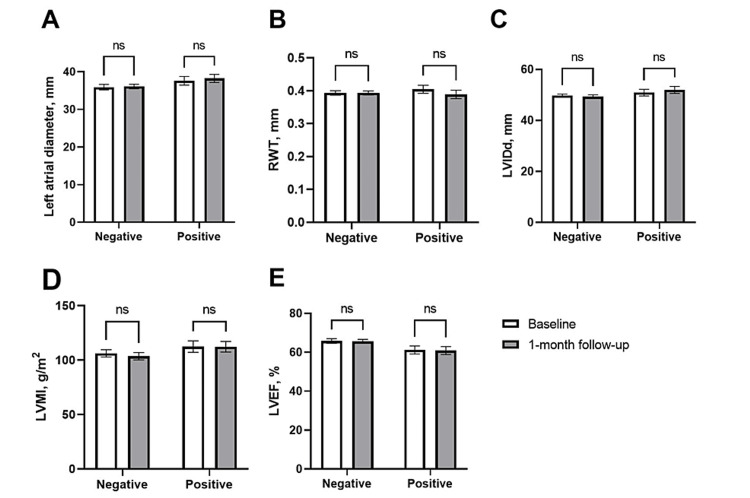
Comparison of echocardiographic parameters in patients between baseline and one-month follow-up after COVID-19. RWT = relative wall thickness; LVIDd = left ventricular internal diameter in diastole; LVMI = left ventricular mass index; LVEF = left ventricular ejection fraction; ns = non-significant

The effect of COVID-19 infection on short-term cardiac injury was determined by comparing the baseline and one-month follow-up echocardiographic parameters in patients with and without COVID-19 infection. As shown in Figure 2, there were no significant changes in echocardiographic parameters, such as left atrial diameter, left ventricular internal diameter in diastole (LVIDd), RWT, left ventricular mass index (LVMI), or LVEF in the negative and positive groups between baseline and one-month follow-up (p > 0.05 for all).

## Discussion

Myocardial injury is common in COVID-19-infected patients and contributes to poor outcomes [4]. Echocardiography is a useful and widely available clinical tool to assess cardiac structure and function. Earlier studies showed that patients infected with COVID-19 were frequently associated with impaired cardiac function assessed by echocardiography [5-7,10]. Further, echocardiographic cardiac abnormality in COVID-19-infected cases was associated with adverse outcomes [6,7,17]. However, no studies compared the differences in clinical and echocardiographic parameters between acute COVID-19-infected and non-infected patients, especially for the Omicron variant. Further, few studies evaluated the association between baseline echocardiographic markers and acute COVID-19 infection. In addition, little is reported about the short-term echocardiographic changes after the Omicron variant of COVID-19 infection.

There is heterogeneity in the association between clinical parameters and the risk of COVID-19 infection. In hospitalized and community-dwelling populations in the United States, cardiovascular diseases were significantly associated with a higher odds rate of COVID-19 infection [1,2]. However, a meta-analysis including patients from China showed that cardiovascular metabolic diseases did not increase the susceptibility to COVID-19 infection [18]. In a community-dwelling population, younger adults and females were reported to have higher COVID-19 rates [1]. However, other studies demonstrated that both older age and male sex were associated with higher COVID-19 rates and poor outcomes [3,19-21]. Here, we observed that patients infected with COVID-19 were older and had higher rates of high blood pressure and heart failure. Further, we found that male gender was associated with higher odds of COVID-19 infection. Higher pulmonary cells expressing angiotensin-converting enzyme 2 (ACE2) in males than females may contribute to these sex differences because ACE2 facilitates COVID-19 invasion [22]. Differences in study population, disease severity, and differences in COVID-19 variants may contribute to the above-mentioned differences. Further studies are required to clarify the association between clinical parameters and COVID-19 infection.

Previous studies reported that COVID-19 infection was associated with acute echocardiographic cardiac dysfunction [23]. These studies were mainly conducted among patients who were already infected with or recovered from COVID-19 [8-11]. However, the association between baseline echocardiographic parameters and COVID-19 infection among hospitalized patients remains less clear, especially for the Omicron variant. In this study, we found that the left ventricular systolic and diastolic functions were relatively normal, which means mild COVID-19. Further, significantly lower LVEF and higher A-wave velocity were observed in the COVID-19-infected group compared with the controls. These differences were recorded at the onset of COVID-19 infection, which reflected a background subclinical echocardiographic cardiac dysfunction. Our study suggested that echocardiographic parameters, such as LVEF and A-wave velocity, may be used to predict future COVID-19 risk.

There is inconsistency regarding the long-term echocardiographic change in COVID-19-infected patients. Early studies conducted in those infected with COVID-19 found that both left and right ventricular function were impaired during the three-month follow-up, especially in severe and moderate cases [24]. However, other studies conducted during the first and second waves showed no significant changes in long-term follow-up (four months to one year) echocardiographic parameters of left or right ventricular in COVID-19-infected patients 25,26]. One study conducted in hospitalized COVID-19 cases found no changes in one-month follow-up echocardiographic parameters in fully vaccinated patients [7]. Similarly, our study found no significant change in echocardiographic parameters during the one-month follow-up. It has been suggested that disease severity, cardiac injury during the acute phase, and vaccination status may influence the results; hence, more studies are needed.

There are several limitations of this study. First, the left ventricular global longitudinal strain (GLS) was not analyzed. It has been reported that left ventricular GLS is more sensitive and can detect subclinical cardiac dysfunction in COVID-19-infected cases, especially in the mild population [8-10]. Second, the right ventricular was not measured. It has been reported that echocardiographic right ventricular abnormality was common and correlated with poor outcomes [27-29]. Lastly, the retrospective design prevented us from determining the causal relationship between baseline clinical and echocardiographic parameters and COVID-19 infection.

## Conclusions

In this single-center retrospective study, we demonstrated that patients infected with COVID-19 were older, were more likely to be male, and had higher hypertension and heart failure rates. Further, echocardiographic parameters such as LVEF and A-wave velocity were independently associated with higher odds of COVID-19 infection. Lastly, COVID-19 infection did not significantly alter echocardiographic parameters in the short-term follow-up period. To our knowledge, this is the first report to investigate the association between baseline clinical and echocardiographic parameters and COVID-19 infection in hospitalized patients in China.
